# Supplementary material for: Dynamics of nitrogen and phosphorus accumulation and their stoichiometry along a chronosequence of forest primary succession in the Hailuogou Glacier retreat area, eastern Tibetan Plateau
Source: PLoS One. 2021 Feb 2;16(2):e0246433. doi: 10.1371/journal.pone.0246433 (PMC7853510; doi:10.1371/journal.pone.0246433)
Supplement: Attachment — Submitted filename: PONEReveiw_1Oct2020.pdf [file pone.0246433.s005.pdf]

Reviewer: Deane Wang (1 Oct 2020)

Manuscript Number: PONE-D-20-29430
Manuscript Title: Dynamics of nitrogen and phosphorus accumulation and their stoichiometry along a chronosequence of succession forests in the Hailuogou Glacier retreat area, eastern Tibetan Plateau

Yang et al.

SUMMARY COMMENT

This article complements a two decades long series of research reports on the Hailuogou glacier retreat area and an associated 125 year chronosequence of ecosystem development. The information provided builds on prior research and adds an analysis of N:P ratios in soils and vegetation to document the changes in nutrient accumulation over the chronosequence. The methods, results and many of the interpretations seem appropriate. The conclusion that N limits plant growth, however, is not fully supported. The authors should more fully defend this assertion, or simply suggest that an N limitation hypothesis is favored by them. The N:P data are important descriptions for this primary successional sequence and are deserving of publication.

I recommend publication with some substantial revisions.

GENERAL COMMENTS

- ABSTRACT/INTRODUCTION

The abstract and introduction should emphasize the same important points. The abstract makes assertions about the role of the N:P ratio in limiting vegetation growth, while the third objective listed at the end of the introduction just list the desire to report on changes in the N:P ratio. It seems like the overriding goal is to better understand nutrient controls on ecosystem development through the description of N:P ratios, with the specific goal of assessing limiting factors.

This work is part of a series of papers on the Hailuogou glacier retreat area, which should be acknowledged and cited in the Introduction. This helps the reader understand the context of the research, and may also help understand what work was done in connection with related research and what work was conducted specifically to answer the N:P ratio questions.

- METHODS

A key paper to describe vegetation methods might be:

Yang D, Luo J, She J, Tang R (2015) Dynamics of vegetation biomass along the chronosequence in Hailuogou glacier retreated area, Mt. Gongga. Ecology and Environmental Sciences 24:1843–1850 (in Chinese with English abstract)

However, I could not access this paper. If the chronosequence and the biomass sampling are the same, then perhaps this material could be included in the supplementary material. (I could also not access the supplementary material for this paper - Yang et al.).

Currently, the Methods section omits much detail, and if this is because the work was related to other work, this should be described (and cited). If, for example, the sampling was done specifically for this paper, then a great deal more information needs to be provided.

For example, were the tree subsamples and the allometric equations developed from the same trees? If the vegetation was sampled for this research, how were the replicate samples distributed within a site? Were they randomly selected? Were shrub and ground cover samples nested within the tree samples? Were the soil samples nested in any of the other sample locations? The high spatial variability of soils and vegetation is generally a challenge for ecosystem research and some acknowledgement of this and how this team approached this problem is useful for future investigators.

The context of this research is also a bit puzzling relative to:

Yang et al. (2014) Dynamics of carbon and nitrogen accumulation and C:N stoichiometry in a deciduous broadleaf forest of deglaciated terrain in the eastern Tibetan Plateau. Forest Ecology and Management 312: 10–1.

That work indicates its chronosequence location as "This study was conducted in the foreland of the Hailuogou glacier (101.99°E, 29.57°N, 2990 m a.s.l.)"

"the whole successional chronosequence, the Populus purdomii forest acts as the broadleaf deciduous stage, which is a band in the valley bottom at elevation from 2844 m to 2950 m."

This paper (under review) also list the elevation: "... forest primary succession sequence of approximately 2 km was formed in the Hailuogou glacier retreat area at an altitude of 2800–2970 m “with similar Lat/Long coordinates. If these chronosequences are the same, the Methods and perhaps the Introduction, should indicate this.

Because the C:N stoichiometry over a chronosequence would require essentially the same methods as a N:P stoichiometry, I would think that these were related studies. This should be clarified.

Were the biomass estimates separate estimates from that of Yang et al. (2014)? If so, some comparison with those biomass and N accumulation rates should be presented in the Results and Discussion.

The regression statistics used (line 161) do not seem like they are appropriate for the statements made about the slope being different from one. Without the data, I can only do a visual estimate, but the significance level of $p<0.001$ seems inappropriate for the presented data, especially for tree, herb and the A layer. A more detailed statistical explanation is needed.

- RESULTS and DISCUSSION

Many factors limit vegetation growth, both individually and in concert with each other. Moisture, sunlight, temperature, nutrients, etc. can result in the observed rate of biomass accumulation. The reported rates of biomass accumulation across this chronosequence seem comparable with other estimates of forest growth rates in similar climates. These glacial ecosystems do not appear to be growth-limited in any unusual ways. Accepting that this study seeks only to examine N and P limitations, it is a fair question to wonder about N vs. P limitations. However, I think it is useful for the authors to present this context of many limitations, and their particular interest in N and P, to their readers.

As an alternative interpretation to their conclusion of N limitation, I might suggest that the accumulation of N in the soil demonstrates the build up of "extra" N. "Tight" cycling of N has been demonstrated in many forested ecosystems, such that the pool of organic N in the forest floor and the soils remains relatively constant over successional time. The absence of this "tight" cycling of N in this primary succession could be interpreted as an indication of the relative availability of N. P on the other hand, is much more stable over the chronosequence in the litter, O and A layers, possibly suggesting that the continuing requirement of P by accumulating biomass takes up any new P from decomposing organic matter while accessing the continuing release of P from primary (and possibly secondary) minerals to fine roots and mycorrhizae.

In the Discussion the authors state:

"Generally, a N:P ratio <14 in leaves indicates that plant growth is limited by N, whereas a N:P ratio >16 indicates that plant growth is limited by P." (line 307)

So it is curious that they do not report their own N:P ratio in leaves as a comparison. Total vegetation N:P ratios are a weaker absolute indicator of nutrient limitations due to the various N:P ratios in different species and different vegetation tissues. They cite (line 311) a reported range from "<10 for N limitation" to ">20 for P" across a broad range of vegetation. Their reported ratio is just at the boundary of 10, which does not make a strong case for N limitation.

Line 41 of the Abstract states: "... N was the main limiting factor for plant growth in this sequence." Given that an abstract may be the only part of the research that some scientists may read, I would suggest that this conclusion needs to be stated more carefully, perhaps with reservations.

SPECIFIC COMMENTS

line 98: If an actual average can not be calculated from recorded data, then two to four significant figures are not needed to give the reader an approximate estimate for temperature and rainfall. In this case ~4 Deg C and ~2000 mm rain would suffice.

line 120: all of the numbers in Table 1 should include at most two significant figures. The estimates are not more precise than that, and having fewer digits to examine makes it easier for the reader.

line 121: Tree biomass was estimated using the allometric equations reported by Liu (2019). Are these the same equations reported by:

X Zhong, N Wu, J Luo, K Yin, Y Tang, Z Pan - Chengdu Science and Technology …, 1997. Researches of the forest ecosystems on Gongga Mountains.

line 125: The difference between "herbs" and "ground cover" is not clear.

line 143: "The thickness and bulk density of each soil layer were then measured using a measuring tape and a cylindrical tube, respectively."

Soil is notoriously hard to sample because of the inherent spatial variation. It would be helpful to include the N (sample size at each site). Table 1 also needs to indicate the unit of the variation (one standard deviation, one standard error (standard deviation of the mean), two standard errors?). Pit sampling (TG Huntington, DF Ryan, et al. 1988 Estimating soil nitrogen and carbon pools in a northern hardwood forest ecosystem. in Soil Science Society of Am.) provides a more accurate bulk density and nutrient concentration estimate.

For ease of examining the data, it would be very helpful to use two/three significant figures, especially given the high variation that seems apparent, e.g. 120.80 +- 13.89 should probably be reported as 120 +- 14; 6.43 +-0.53 is probably best as 6.4 +- 0.5.

line 174: "vegetation N pool sharply increased" The word "sharply" does not seem appropriate here as the accumulation rate of N over the 125 years appears linear. "Sharply" is used again on line 229.

line 201: The rates of relative N and P changes in Figure 3 are not consistent with the explained methods. Equation (1) line 157 shows the calculation of rate of relative change for each site (i = 1 to 6). Figure 3 shows more than 6 points. The construction of Figure 3 needs to be explained.

line 202: The statistics associated with Figure 3 indicate a significant correlation between N and P (i.e. a slope significantly different than 0). However, in order to make a statement about the importance of the slope being different than 1.0, the 95% confidence bounds on the slope estimate (e.g. slope of 1.26 for trees) needs to be provided. Also Figure 3 shows a slope of 1.26

while line 206 states a slope of 1.32. Other inconsistencies between the text and Figure 3 are also presented.

line 299: "also showed that the rate of relative N accumulation was faster than that of P in surface soil." Without knowing if the reported slopes were significant or not (see comment above, line 202), the results of this study may only "suggest." However, the point here should probably be about which processes are causal and which observations are just incidental to those processes. A higher relative N accumulation rate WILL result in a change in N:P ratio, in all cases. The more salient question is how N and P cycling are changing (relative to each other) as the forest stand matures.

line 301: In this primary successional sequence it seems unlikely that P has weathered out of the rocks and is less available at the end of the chronosequence.

line 316: "owing to the high N level of N-fixing" adding "perhaps due to" would be a fairer statement of likelihood. This study did not establish the biogeochemical role of N-fixing microbes and plants at these sites. Making a causal claim about what particular biogeochemical process alters the relative uptake of nutrients is therefore not appropriate as a statement, and more appropriate as an interpretation or hypothesis.

line 326: "This increase in N and decrease in P may shift the factor limiting plant growth from N to P after several hundred years. Therefore, the dynamics of the N:P ratio in this forest primary succession needs further study." Successional changes in relative nutrient availability are important to document, and this 125 year sequence provides a good example of nutrient dynamics in a chronosequence. However, the N limitation assertion is an hypothesis, and the authors should be careful in how they state their interpretations. Given the various ratios of N:P they report at their sites, the N limitation is possible, but not conclusive.

line 339: "whereas the N:P ratio in vegetation maintained a constant low level due to the tree layer having a rapid P accumulation rate compared with N;" "higher" rather than "rapid" is probably more appropriate here. Plants and ecosystem accumulate nutrients according to their needs, and we can only infer limitation if we do not determine this experimentally (e.g. with a nutrient addition experiment).
